# Effectiveness of oral semaglutide on glucose control and body weight up to 18 months: a multicenter retrospective real-world study

**DOI:** 10.1007/s40618-024-02309-2

**Published:** 2024-02-18

**Authors:** B. M. Bonora, G. Russo, F. Leonetti, M. Strazzabosco, L. Nollino, G. Aimaretti, A. Giaccari, F. Broglio, A. Consoli, A. Avogaro, G. P. Fadini, For the GLIMPLES Study Investigators

**Affiliations:** 1 Department of Medicine, University of Padova, Via Giustiniani 2, 35128 Padua, Italy; 2 Veneto Institute of Molecular Medicine, Padua, Italy; 3 Department of Clinical and Experimental Medicine, University of Messina, Messina, Italy; 4 Department of Medical and Surgical Sciences and Biotechnologies, Sapienza University of Rome, Rome, Italy; 5 Diabetology and Metabolic Diseases Unit, S. Bortolo Hospital, Vicenza, Italy; 6 Department of Medicine, Diabetology Service, Azienda ULSS 2 Marca Trevigiana, Treviso, Italy; 7 Endocrinology, Department of Translational Medicine, Università del Piemonte Orientale, Novara, Italy; 8 Centro per le Malattie Endocrine e Metaboliche, Fondazione Policlinico Universitario A. Gemelli IRCCS and Università Cattolica del Sacro Cuore, Rome, Italy; 9 Division of Endocrinology, Diabetes and Metabolism, Department of Medical Sciences, University of Turin, Turin, Italy; 10 Endocrinology and Metabolism Unit, ASL, Pescara, Italy; 11 Department of Medicine and Aging Sciences DMSI and Center for Advanced Studies and Technology CAST, “G. D’Annunzio” University of Chieti-Pescara, Chieti, Italy

**Keywords:** Oral semaglutide, Type 2 diabetes, Glucose control, Body weight, Real-world effectiveness, Retrospective study

## Abstract

**Aim:**

Oral semaglutide, an innovative orally administered GLP-1 receptor agonist for type 2 diabetes (T2D) management was herein evaluated for its effectiveness in a multi-center retrospective real-world study.

**Methods:**

We included new-users of oral semaglutide from 18 specialist care centres and collected retrospective data on baseline clinical characteristics. Updated values of HbA1c and body weight were analyzed using the mixed model for repeated measures.

**Results:**

The study included 166 individuals with T2D, predominantly men (64.5%), with a mean age of 64.4 years and a mean diabetes duration of 10.1 years. In the majority of patients (68.3%) oral semaglutide was used as a second-line drug, mostly with metformin. At baseline, mean BMI was 28.9 kg/m^2^ and HbA1c was 7.5%. During the 18-month observation period, oral semaglutide demonstrated significant reductions in HbA1c, with a maximum change of − 0.9%, and 42.1% of patients achieved HbA1c values below 7.0%. Additionally, there was a substantial reduction in body weight, with an estimated change of − 3.4 kg at 18 months, and 30.3% of patients experienced a 5% or greater reduction in baseline body weight. Only 24.2% of patients reached the 14 mg dose. Subgroup analysis revealed that baseline HbA1c > 7%, persistence on drug, not being on a prior therapy with DPP-4 inhibitors, and loosing 5% or more the initial body weight were associated with greater HbA1c reductions.

**Conclusion:**

This study supports oral semaglutide as an effective option for T2D treatment, offering improved glucose control and weight management in a real-world setting.

## Introduction

Oral semaglutide stands out as the pioneering orally administered peptide hormone-based treatment for type 2 diabetes (T2D). It harnesses advanced pharmaceutical techniques to ensure efficient absorption and efficacy when taken orally [1]. Unlike injectable GLP-1 receptor agonists (GLP-1RA), it offers the benefit of increased patient acceptance [2].

The findings from the PIONEER trial program establish oral semaglutide as the most effective medication for controlling blood glucose levels and body weight among existing oral T2D treatments [3]. Furthermore, it exhibits potential in improving cardiovascular risk indicators such as blood pressure, lipids, abdominal fat, and inflammation [4–6]. These attributes position oral semaglutide as a favorable choice for early T2D treatment when metformin monotherapy fails or is contraindicated [3].

Although concrete evidence regarding its effect on cardiovascular outcomes is pending, it is worth noting that the same active compound, when given subcutaneously in the SUSTAIN-6 trial, reduced the occurrence of major adverse cardiovascular events (MACE) compared to a placebo [7]. In the pre-marketing PIONEER-6 trial, a similar reduction in MACE rates was observed for oral semaglutide versus placebo, though statistical significance was not reached due to smaller event numbers than in SUSTAIN-6 [8]. Nevertheless, there were notable reductions in overall mortality and cardiovascular-related deaths with oral semaglutide compared to a placebo [8]. The ongoing SOUL trial is further evaluating the rates of cardiovascular events in individuals with T2D receiving oral semaglutide or placebo [9].

Previous studies with injectable GLP-1RA indicate their use in later stages of T2D, often in patients with a higher prevalence of cardiovascular disease and concurrent insulin therapy [10]. The potential placement of oral semaglutide in the earlier stages of T2D management remains uncertain. Such a shift could introduce a new approach to address therapeutic inertia and improve the likelihood of attaining and maintaining treatment goals in T2D. Recently, we have analyzed the data of 4449 patients deemed to be candidate for initiation of oral semaglutide: the population had a relatively short disease duration (42% < 5 years), and a minority (15.6%) had a history of cardiovascular event, supporting the potential of early implementation of oral semaglutide as a strategy to overcome therapeutic inertia and enhance T2D management [11]. Projected glycemic effectiveness analysis based on trial data revealed that oral semaglutide could potentially lead HbA1c to target in > 60% of patients [11], but there are still limited real-world studies on the effects of oral semaglutide on HbA1c and body weight in clinical practice. The IGNITE study (*n* = 782) reported a reduction in HbA1c of 0.9% from a baseline of 8.4%, but more than one in three patients received only the 3 mg dose [12]. Similar results were obtained in a case-series of patients treated with oral semaglutide for 6 months, reporting a HbA1c reduction of 0.8% from a baseline of 8.2%, with a strong response among those with a starting HbA1c > 9% [13]. Candido et al. analyzed the data of 129 patients with T2D who initiated oral semaglutide: after 6 months with 29% of patients reaching the 14 mg dose, HbA1c declined from 7.2% to 6.9% and body weight declined by 2.0 kg [14]. In a smaller studies conducted in Japan, patients receiving oral semaglutide experienced a reduction in HbA1c of 1.2% and in body weight of 1.4 kg, with a mean dose slightly above 7 mg [15]. While these data are overall re-assuring on the effectiveness of oral semaglutide in clinical practice, there is still a lack of long-term studies and continuous post-marketing monitoring is warranted.

We herein present results of a multicenter retrospective study on new users of oral semaglutide with an observation up to 18 months, providing new data on effectiveness on glucose control and body weight.

## Methods

### Study design

GLIMPLES (GLp-1 for therapeutic sIMPLification in type 2 diabetES) was a multicenter retrospective study promoted by the Italian Diabetes Society and conducted in 18 diabetes specialist care centers in Italy. Because, in Italy, only diabetes specialists could initiate therapy with GLP-1RA during the data collection period, the population under investigation is supposed to be representative of the entire population of patients who initiated GLP-1RA in the Italian clinical practice at the time.

The study was first approved by the Ethical Committee of the coordinating center (University Hospital of Chieti-Pescara, prot. n. 09 dated 19/05/2022) and by the Ethical Committees of all participating Centers, and conducted according to the principles of the Declaration of Helsinki. Data were extracted automatically from the same electronic chart system (MetaClinic, Me.Te.Da, San Benedetto del Tronto, Italy) at all centers. In compliance with the national regulation on observational retrospective studies on routine care data (Det. AIFA 20/03/2008) and in agreement with the national and European (GDPR) privacy policy, data were anonymized at time of extraction from the chart system and the need for informed consent was waived. Anonymization was performed according to the guidance set forth in [16]. Specifically, the following procedures were applied by the data extraction software: (i) avoiding to collect personal information (including name, surname, patient id, date of birth, place of birth, address); (ii) applying differential privacy (i.e., collecting only the data that were deemed to be needed to conduct the study); (iii) generalizing and aggregating variables; (iv) introducing random error to all continuous variables, with a tolerance range decided in advance for each variable. These operations were designed to meet a trade-off between ensuring reasonable irreversibility of anonymization and limiting the impact on the scientific validity of the data being collected.

### Definition of exposure

The study collected baseline and follow-up data on all patients that initiated for the first time a GLP-1RA between 01/01/2010 and 31/12/2021. Initiation of GLP-1RA was defined as a new prescription of any GLP-1RA available in Italy (exenatide BID, exenatide OW, lixisenatide, liraglutide, dulaglutide, semaglutide OW, oral semaglutide) for patients who had never been treated with the same compound before, as evident from the diabetes treatment history recorded in the electronic chart. With this definition, we also collected data of patients switching from one GLP-1RA to another. The index date was set as the date patients were prescribed the index drug. The database contained no information on drug dispensation or refill rates, so that it was impossible to calculate medical possession ratio or adherence. Persistence on treatment was defined based on refilled prescription at follow-up visits.

In the present study, we analyzed only data on new-users of oral semaglutide, with the aim of describing effectiveness on HbA1c and body weight. There was no control group. Inclusion criteria were: age 18 years or older, a diagnosis of type 2 diabetes done at least one year before index date as recorded in the chart; initiation of oral semaglutide on the background of any therapeutic regimen including diet alone; no treatment with any other GLP-1RA in the prior visit. Exclusion criteria were: age < 18; diabetes other than type 2; lack of follow-up data in the electronic chart at least for one of the endpoints described below; switch from a different GLP-1RA.

### Variables

At baseline, we collected the following information for all patients: age, self-reported gender, diabetes duration, body weight, height, body mass index, systolic and diastolic blood pressure, fasting glucose, HbA1c, lipid profile (LDL cholesterol was calculated from total cholesterol, HDL cholesterol and triglycerides using the Friedewald equation), the estimated glomerular filtration rate (eGFR) calculated from serum creatinine using the CKD-EPI equation, and urinary albumin/creatinine ratio (UACR). We also collected information on the following chronic complications of diabetes: stage III or higher chronic kidney disease (CKD), defined as an eGFR of 60 ml/min/1.73 m^2^ or less; pathologic albuminuria (UACR > 30 mg/g), retinopathy (any grade, according to digital fundus examination scored by expert ophthalmologists); coronary heart disease (CHD) defined as either myocardial infarction, angina, coronary revascularization or instrumental evidence of cardiac ischemia; established CVD, including CHD or stroke or any site revascularization; any microangiopathy (including diabetic kidney disease, retinopathy, or neuropathy), any macroangiopathy (including any grade of asymptomatic atherosclerosis in the coronary, carotid or peripheral circulation). Finally, we collected information on concomitant medications for the management of diabetes and cardiovascular risk factors. The dose of oral semaglutide at each visit was recorded along with the information on whether the prescription was confirmed or not.

### Endpoints

The primary study endpoint was the change in HbA1c from baseline through follow-up visits with confirmed prescription for oral semaglutide. Secondary endpoints were: the change over time in body weight; the change in systolic blood pressure and total cholesterol at 6 months; the proportion of patients achieving a HbA1c < 7% at any observation among those with baseline HbA1c > 7%; the proportion of patients achieving > 5% body weight reduction at any observation compared to baseline.

### Statistical analysis

Descriptive statistics are presented as mean (standard deviation) for continuous variables or as percentages for categorical variables. Comparison between continuous variables were performed with the Student’s t test, whereas categorical variables were compared using the chi square test. The change over time in HbA1c and body weight were estimated using the mixed model for repeated measures. Time, baseline HbA1c and dose of oral semaglutide were entered as fixed factors. The unstructured covariance was used and the model output were estimated means at each time point along with 95% confidence interval. We also calculated the greatest HbA1c reduction for each patient, which was used for a stratified analysis: patients were divided according to a few key baseline characteristics and the greatest HbA1c reduction was compared across strata to evaluate if any patient subgroup displayed greater glycaemic improvements. The same analysis was repeated for body weight. Statistical significance was accepted at the conventional 5% type 1 error. SPSS version 23 was used for all analyses.

## Results

### Patient population

We included 166 individuals who initiated oral semaglutide and had at least one available follow-up examination after baseline (Table 1). Participants (64.5% men) had a mean age of 64.4 years and a mean diabetes duration of 10.1 years. Baseline BMI was 28.7 kg/m^2^ and HbA1c was 7.5% (58 mmol/mol). Regarding the complication burden, 34.6% of patients had at least one microangiopathy and 49.2% had macroangiopathy. Baseline eGFR was 84.5 ml/min/1.73 m^2^, 11.9% had CKD stage III or higher, and 22.0% had micro- or macroalbuminuria. At the time of initiation of oral semaglutide, 92.8% of patients were on metformin, 21.1% were on SGLT-2 inhibitors and 7.2% on a sulphonylurea. There was a small percentage (6.0%) of patients concomitantly treated with basal insulin. Patients switching from an injectable GLP-1RA based regimen were excluded, but 30.2% were previously treated with a DPP-4 inhibitor. In 68.3% of patients, oral semaglutide was being used as a second-line diabetes drug.

**Table 1:** Characteristics of study patients

	Available (%)	Value
Demographics		
Male sex, %	100.0	64.5
Age, years	100.0	64.4 (8.6)
Diabete duration, years	100.0	10.1 (8.2)
Clinical and laboratory data		
Body weight, kg	84.3	81.7 (16.9)
Body mass index, kg/m^2^	83.1	28.9 (5.3)
Systolic blood pressure, mm Hg	71.7	141.2 (19.5)
Diastolic blood pressure, mm Hg	71.7	80.0 (11.0)
Fasting glucose, mg/dl	89.8	153.3 (46.3)
HbA1c, %	94.6	7.5 (1.3)
HbA1c, mmol/mol	94.6	58.4 (14.2)
Total cholesterol, mg/dl	80.7	165.4 (38.6)
HDL cholesterol, mg/dl	81.3	49.8 (13.7)
LDL cholesterol, mg/dl	78.3	89.2 (31.7)
Triglycerides, mg/dl	81.3	138.5 (75.3)
eGFR, ml/min/1.73 m^2^	86.1	84.5 (18.4)
UACR, mg/g	65.7	39.8 (80.5)
Complications	97.6	
CKD stage III + , %		11.9
Albuminuria > 30 mg/g, %		22.0
Retinopathy, %		17.2
Coronary heart disease, %		14.7
Established CVD, %		16.9
Any microangiopathy, %		34.6
Any macroangiopathy, %		49.2
Diabetes medications	100.0	
Metformin, %		92.8
Sulphonylureas, %		7.2
Semaglutide, %		100.0
SGLT-2 inhibitors, %		21.1
Pioglitazone, %		4.2
Basal insulin, %		6.0
Other therapies	100.0	
Statin, %		71.6
Anti-platelet agents, %		33.8
RAS blockers, %		56.1
Beta-blockers, %		26.4
Calcium channel blockers, %		20.9
Diuretics, %		19.6
Anticoagulants, %		2.7

Data are presented as mean (standard deviation) or as percentage. Percent availability is indicated. eGFR, estimated glomerular filtration rate. UACR, urinary albumin excretion rate. CKD, chronic kidney disease (defined as eGFR < 60 ml/min/1.73 m^2^). SGLT-2, sodium glucose co-transporter-2. RAS, renin angiotensin system

### Dose, duration and persistence of treatment

**Fig. 1:**
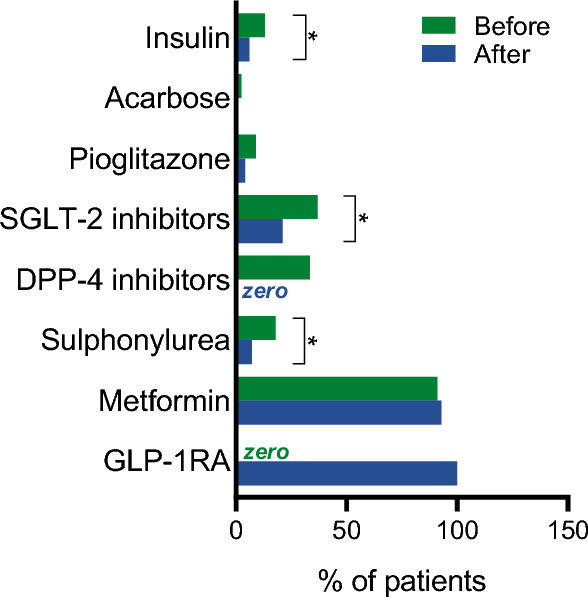
Change in concomitant glucose lowering medications. The frequency of glucose lowering medications classes is reported for the visit before initiation of oral semaglutide and at the time of initiation. *p < 0.05

The recommended timing for taking oral semaglutide was pre-breakfast in 86.1% of cases. As compared to the prior regimen, initiation of oral semaglutide was associated with a significant reduction in the concomitant use of DPP-4 inhibitors (dropping to zero as expected), sulphonylureas, SGLT-2 inhibitors, and insulin (Fig. 1).

The median follow-up time was 12.5 months (IQR 8.6–15.9), with a maximum span of 2 years. During observation, prescription of oral semaglutide was discontinued in 39.4% of patients. Based on Kaplan–Meier curve analysis, the estimated median time on drug was 19.3 months (IQR 16.8–20.5). The last dose of oral semaglutide was 3 mg in 7.3% of patients, 7 mg in 68.5% of patients and 14 mg in 24.2% of patients. These proportions were similar in those who discontinued treatment (10.7%, 78.8% and 10.6%, respectively). The most common regimens initiated after discontinuation of oral semaglutide were: injectable GLP-1RA (11.4% dulaglutide, 9.6% semaglutide, 1.8% liraglutide) and SGLT-2 inhibitors (35.3%). Baseline HbA1c was lower among those who discontinued treatment with oral semaglutide during the observation, compared to the rest of the cohort (7.2% vs 7.7%; *p* = 0.03).

### Glycaemic control and body weight change

**Fig. 2:**
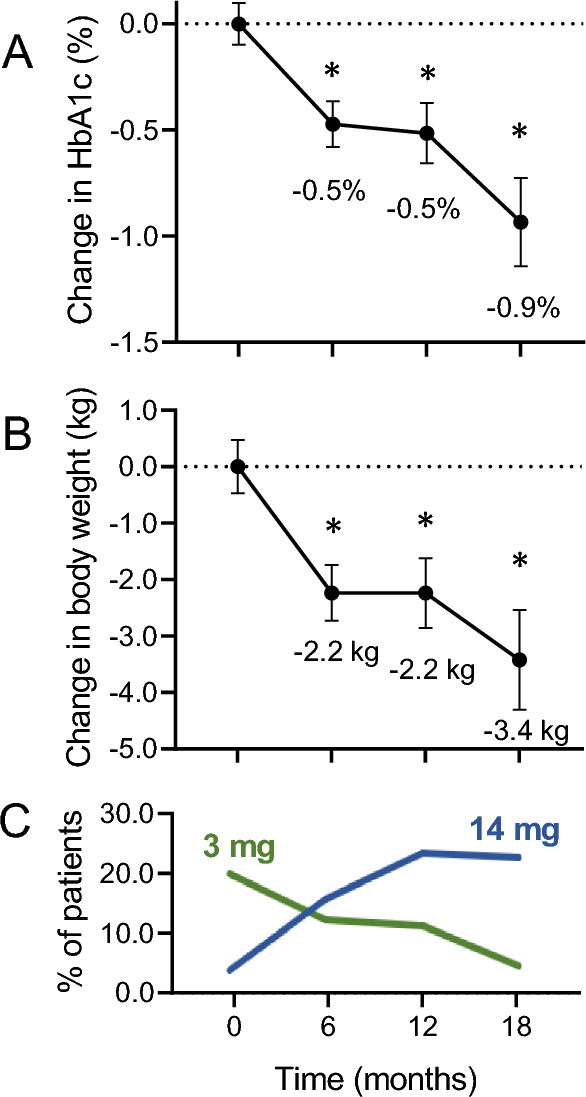
Effectiveness on HbA1c and body weight. **A** The change in HbA1c from baseline to 18 months (*p < 0.05 versus baseline). **B** The change in body weight from baseline to 18 months (*p < 0.05 versus baseline). **C** The change in percent patients using the 3 mg and the 14 mg dose (all the other patients were prescribed the 7 mg dose at each timepoint)

The change over time in HbA1c and body weight was estimated using the mixed model for repeated measures, which considers all patients contributing with at least one value at baseline or during observation, until discontinuation of oral semaglutide. The maximum estimated change in HbA1c, adjusted for baseline HbA1c and drug dose, was -0.9% (0.2) at 18 months (Fig. 2A). During the observation, 42.1% of patients who had a baseline HbA1c above 7.0% achieved an HbA1c value below 7.0%. Among patients who discontinued treatment 47.5% did not achieve a reduction of HbA1c compared to baseline.

The estimated change in body weight, adjusted for baseline weight and drug dose, was − 3.4 kg (0.8) at 18 months (Fig. 2B) and 30.3% of patients experienced a body weight reduction of 5% or greater the baseline body weight.

We found a statistically significant, but weak, direct correlation between the change in HbA1c and the change in body weight (*r* = 0.22; *p* = 0.004): weight loss would explain less than 5% of the magnitude of the change in HbA1c.

At 6 months after index date, systolic blood pressure significantly declined by 6.2 mm Hg (95% C.I. − 10.7 to − 1.8 to mm Hg) and total cholesterol significantly declined by 14.4 mg/dl (95% C.I. − 21.0 to − 7.7 mg/dl).

### Subgroup analysis

**Fig. 3:**
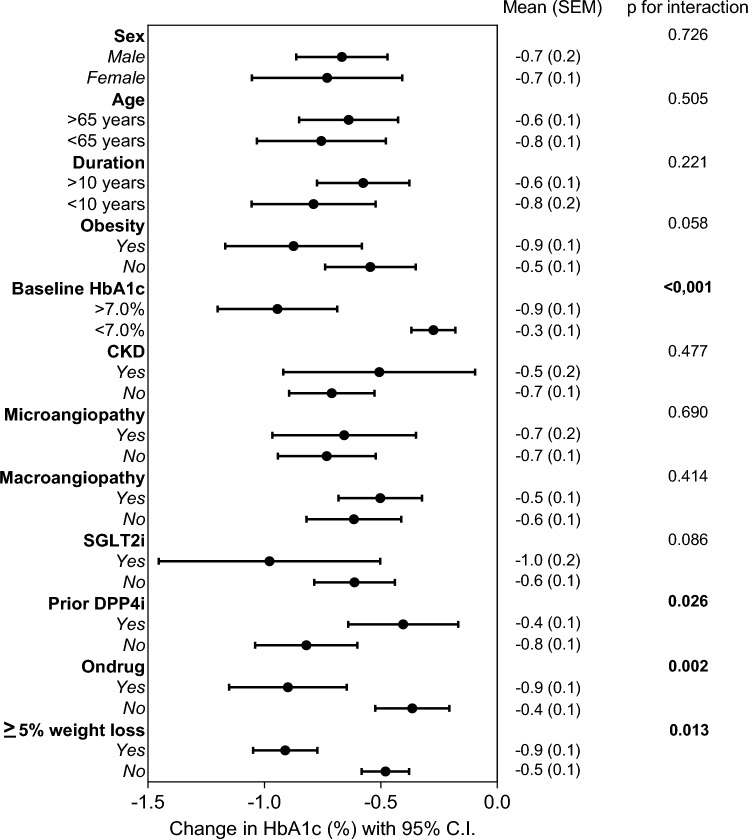
Subgroup analysis for the effect on HbA1c. The patients were divided into strata according to some key clinical features at baseline: the forest plot shows the change in HbA1c in each stratum along with 95% C.I. and the *p*-value for the between-group comparison

We stratified patients by a series of baseline variables and compared the greatest reduction in HbA1c experienced by the patients within each stratum during observation, irrespective of whether the drug was discontinued. Baseline HbA1c > 7%, persistence on drug and not being on a prior therapy with DPP-4 inhibitors were associated with significantly greater HbA1c reduction. No heterogeneity of the glycaemic response was observed according to age (< > 65 years), diabetes duration (< > 10 years), BMI (< > 30 kg/m^2^), presence of CKD stage III or higher, microangiopathy, macroangiopathy, or concomitant treatment with SGLT-2 inhibitors (Fig. 3). None of such stratification variables was associated with different changes in body weight.

The improvement in HbA1c was twice as large in patients who, during observation, lost 5% or more their initial body weight than in those who did not (− 0.9% vs − 0.5%; *p* = 0.013).

## Discussion

In the Italian specialist diabetes care practice, oral semaglutide was initiated in patients with an average baseline HbA1c of 7.5% and BMI of 29 kg/m^2^. During a median follow-up of roughly one year, HbA1c declined by 0.9% bringing 42% of patients who had HbA1c > 7% down to the conventional HbA1c target of < 7%. Mean body weight reduction was 3.4 kg and 30% of patients experienced a body weight reduction of 5% or more their initial body weight. Baseline HbA1c and persistence on oral semaglutide were factors significantly associated with glycaemic response. The greatest benefit was observed among patients who were naïve to DPP-4 inhibitors, but a significant reduction in HbA1c was noted even after switching from a DPP-4 inhibitor to oral semaglutide. An early improvement in cardiovascular risk factors, like blood pressure and total cholesterol, was also observed after initiation of oral semaglutide.

It is important to underline that these results were achieved with less than the maximal dose of oral semaglutide in the majority of patients. Indeed, at the end of the observation, roughly 2 out of 3 patients were receiving the 7 mg maintenance dose and only 1 out of 4 had escalated to the maximum licenced 14 mg dose. We do not have information on whether this incomplete dose escalation was due to gastrointestinal side effects or a lack of perceived need to further improving glycaemic and body weight control, as several patients reached HbA1c values below 7% with the 7 mg dose. Yet, a small fraction of patients remained on the starting 3 mg dose, which is not considered a maintenance dose of oral semaglutide. In such cases, the reason for not increasing to 7 mg could be related to gastrointestinal side effects or to a lack of patient’s and healthcare professional’s action for dose escalation. In general, it can be anticipated that targeting the maximal 14 mg dose in all patients, as done in RCTs [3], would have allowed even greater improvements in glycaemic control and body weight.

As expected from data on injectable semaglutide [17], we found a weak though significant direct correlation between the improvement in HbA1c and the loss of body weight. The greatest improvement in HbA1c was observed among patients who lost 5% or more their initial body weight, reinforcing the importance of weight management in the achievement of treatment goals for people with T2D [18].

So far, this is the longest real-world study on oral semaglutide. The follow-up of our study was longer than that of the IGNITE international observational study (about 6 months) [12], though further observational studies will be needed to assess the long-term benefits and persistence of glycaemic and body weight effectiveness of oral semaglutide. The importance of dose optimization is also evidence looking at the shape of the HbA1c and body weight curves over time, which tended to plateau between 6 and 9 months, and then declined further after 12 months. Given that, in the Italian specialist care setting, patients with T2D who are not on insulin are seen every 6–9 months, we assume that most patients were recommended to self-titrate to 7 mg after one month of therapy with the 3 mg dose, but that the titration to 14 mg was suggested after the follow-up visit. Therefore, to overcome this inertia, tighter self-titration schedules may be proposed.

The estimated median persistence time on oral semaglutide was 19 months. Previous studies reporting data on persistence on GLP-1RA have been disappointing. An analysis of German data showed a median persistence time of 11 months, with a trend increase from the 2007–2012 to the 2017–2020 period [19]. Although we have no information on the reasons leading to treatment discontinuation, the high proportion of patients not achieving HbA1c reduction among those who discontinued may indicate a lack of efficacy. However, it should be noted that baseline HbA1c was lower (7.2%) in those who subsequently discontinued oral semaglutide, possibly suggesting that the drug was being used not only for glucose control, but also for weight management. After withdrawing therapy with oral semaglutide, in most cases, patients switched to an injectable GLP-1RA or to an SGLT2i, all drugs provided with demonstrated cardiovascular and renal protection [20]. While there is still no evidence that oral semaglutide can protect people with T2D from adverse cardiovascular or renal outcomes, it has been demonstrated that dose-concentration curves of injectable and oral semaglutide can exert consistent cardiovascular effects [21]. In addition, the evidence that oral semaglutide can significantly improve not only glycaemic control and body weight but also cardiovascular risk factors (including blood pressure, lipids and inflammation), supports a cardioprotective action [6]. Waiting for the results of the ongoing SOUL trial, we hypothesize that formal evidence of cardiovascular efficacy could improve the confidence and the persistence on oral semaglutide.

Oral semaglutide obtained reimbursement approval in Italy in July 2021 and we included in this analysis patients who initiated the drug up to December 2021, thereby reflecting the first 6 months of use in clinical practice. This is important because a learning curve always follows the availability of new medications and the initial use may not reflect the subsequent positioning of the drug or the recommended use according to therapeutic algorithms. A relatively low HbA1c level at the time of initiation of oral semaglutide was reported recently by Candido et al. but, in such study, most patients were switching from a regimen containing DPP-4 inhibitors [14]. With the progressive decline in the use of DPP-4 inhibitors [22, 23], the phenotype of patients initiating oral semaglutide may change accordingly. Therefore, generalizability of our findings need to be carefully considered in view of this evolving therapeutic scenario.

With this caveat in mind, overall patient characteristics suggest that oral semaglutide was being initiated in a relatively early stage of the disease. This is not represented by short disease duration, but mainly by the complication burden and the background therapeutic regimen, characterized by a predominance of metformin and a small proportion of insulin users. Indeed, the average number of other glucose lowering medication classes at the time of initiation of oral semaglutide was 1.31, indicating that oral semaglutide was being used mostly in a dual oral regimen. In addition, the prevalence of retinopathy, kidney disease, and established cardiovascular disease were relatively low compared with those observed in the Italian population of patients with T2D under specialist care [22]. Prior data collected between 2010 and 2018 showed that GLP-1RA had been prescribed for patients with progressively more advanced disease stage, frequent use of insulin and high prevalence of cardiovascular disease [10]. Now, the observation that oral semaglutide was being initiated in patients with a relatively low prevalence of complications and use of insulin is reassuring that the oral delivery route is helping re-positioning GLP-1RA earlier in the natural course of T2D.

The study has several limitations. First, the lack of a control groups makes it impossible to dissect the true contribution of oral semaglutide to the glycaemic and body weight reduction from that of lifestyle changes that could have occurred concomitantly. Second, not all patients had the same observation time and schedule of follow-up, resulting in the need to force the data into 6-month intervals and to model HbA1c and body weight changes. Third, we had no information on tolerability, adherence and reasons for discontinuation of oral semaglutide, thereby providing quite limited information on strategies to improve persistence.

## Conclusion

In this real-world study, oral semaglutide, an orally administered GLP-1 receptor agonist, demonstrated effectiveness in managing T2D. Significant reductions in HbA1c levels (-0.9%) and substantial weight loss (− 3.4 kg) were observed over an 18-month period, along with an early improvement in cardiovascular risk factors. While larger and longer studies are needed, these findings endorse oral semaglutide as a suitable option for early T2D treatment, offering both improved glucose control and weight management in clinical practice.

## Data Availability

Original data used for this article are not publicly available due to national privacy regulation. Aggregate data may be available from the corresponding author upon reasonable request.

## References

[1] Buckley ST, Baekdal TA, Vegge A, Maarbjerg SJ, Pyke C, Ahnfelt-Ronne J, Madsen KG, Scheele SG, Alanentalo T, Kirk RK, Pedersen BL, Skyggebjerg RB, Benie AJ, Strauss HM, Wahlund PO, Bjerregaard S, Farkas E, Fekete C, Sondergaard FL, Borregaard J, Hartoft-Nielsen ML, Knudsen LB (2018). Transcellular stomach absorption of a derivatized glucagon-like peptide-1 receptor agonist. Sci Transl Med.

[2] Gallwitz B, Giorgino F (2021). Clinical perspectives on the use of subcutaneous and oral formulations of semaglutide. Front Endocrinol (Lausanne).

[3] Rodbard HW, Dougherty T, Taddei-Allen P (2020). Efficacy of oral semaglutide: overview of the PIONEER clinical trial program and implications for managed care. Am J Manag Care.

[4] Mosenzon O, Capehorn MS, De Remigis A, Rasmussen S, Weimers P, Rosenstock J (2022). Impact of semaglutide on high-sensitivity C-reactive protein: exploratory patient-level analyses of SUSTAIN and PIONEER randomized clinical trials. Cardiovasc Diabetol.

[5] Rodbard HW, Rosenstock J, Canani LH, Deerochanawong C, Gumprecht J, Lindberg SO, Lingvay I, Sondergaard AL, Treppendahl MB, Montanya E (2019). Oral semaglutide versus empagliflozin in patients with type 2 diabetes uncontrolled on metformin: the PIONEER 2 trial. Diabetes Care.

[6] Yanai H, Hakoshima M, Adachi H, Katsuyama H (2022). A significant effect of oral semaglutide on cardiovascular risk factors in patients with type 2 diabetes. Cardiol Res.

[7] Marso SP, Bain SC, Consoli A, Eliaschewitz FG, Jodar E, Leiter LA, Lingvay I, Rosenstock J, Seufert J, Warren ML, Woo V, Hansen O, Holst AG, Pettersson J, Vilsboll T (2016). Semaglutide and cardiovascular outcomes in patients with type 2 diabetes. N Engl J Med.

[8] Husain M, Birkenfeld AL, Donsmark M, Dungan K, Eliaschewitz FG, Franco DR, Jeppesen OK, Lingvay I, Mosenzon O, Pedersen SD, Tack CJ, Thomsen M, Vilsboll T, Warren ML, Bain SC (2019). Oral semaglutide and cardiovascular outcomes in patients with type 2 diabetes. N Engl J Med.

[9] McGuire DK, Busui RP, Deanfield J, Inzucchi SE, Mann JFE, Marx N, Mulvagh SL, Poulter N, Engelmann MDM, Hovingh GK, Ripa MS, Gislum M, Brown-Frandsen K, Buse JB (2023). Effects of oral semaglutide on cardiovascular outcomes in individuals with type 2 diabetes and established atherosclerotic cardiovascular disease and/or chronic kidney disease: design and baseline characteristics of SOUL, a randomized trial. Diabetes Obes Metab.

[10] Fadini GP, Frison V, Rigato M, Morieri ML, Simioni N, Tadiotto F, D'Ambrosio M, Paccagnella A, Lapolla A, Avogaro A (2020). Trend 2010–2018 in the clinical use of GLP-1 receptor agonists for the treatment of type 2 diabetes in routine clinical practice: an observational study from Northeast Italy. Acta Diabetol.

[11] Morieri ML, Candido R, Frontoni S, Disoteo O, Solini A, Fadini GP (2023). Clinical features, cardiovascular risk profile, and therapeutic trajectories of patients with type 2 diabetes candidate for oral semaglutide therapy in the Italian specialist care. Diabetes Ther.

[12] Aroda VR, Faurby M, Lophaven S, Noone J, Wolden ML, Lingvay I (2021). Insights into the early use of oral semaglutide in routine clinical practice: the IGNITE study. Diabetes Obes Metab.

[13] Frazer M, Swift C, Gronroos NN, Sargent A, Leszko M, Buysman E, Alvarez S, Dunn TJ, Noone J, Guevarra M (2023). Real-world hemoglobin A1c changes, prescribing provider types, and medication dose among patients with type 2 diabetes mellitus initiating treatment with oral semaglutide. Adv Ther.

[14] Candido R, Gaiotti S, Giudici F, Toffoli B, De Luca F, Velardi V, Petrucco A, Gottardi C, Manca E, Buda I, Fabris B, Bernardi S (2023). Real-world retrospective study into the effects of oral semaglutide (as a switchover or add-on therapy) in type 2 diabetes. J Clin Med.

[15] Yamada H, Yoshida M, Funazaki S, Morimoto J, Tonezawa S, Takahashi A, Nagashima S, Masahiko K, Kiyoshi O, Hara K (2023). Retrospective analysis of the effectiveness of oral semaglutide in type 2 diabetes mellitus and its effect on cardiometabolic parameters in Japanese clinical settings. J Cardiovasc Dev Dis.

[16] (2021) Veneto Region: Code of conduct for the use of health data for teaching and scientific publication purposes. https://www.garanteprivacyit/home/docweb/-/docweb-display/docweb/9535354. Last access on Oct 2023

[17] McCrimmon RJ, Catarig AM, Frias JP, Lausvig NL, le Roux CW, Thielke D, Lingvay I (2020). Effects of once-weekly semaglutide vs once-daily canagliflozin on body composition in type 2 diabetes: a substudy of the SUSTAIN 8 randomised controlled clinical trial. Diabetologia.

[18] American Diabetes Association Professional Practice Committee (2024) 8. Obesity and weight management for the prevention and treatment of type 2 diabetes: standards of care in diabetes-2024. Diabetes Care 47:S145–S157 10.2337/dc24-S008 PMC10725806 38078578

[19] Jung H, Tittel SR, Schloot NC, Heitmann E, Otto T, Lebrec J, Pavel M, Lanzinger S (2023). Clinical characteristics, treatment patterns, and persistence in individuals with type 2 diabetes initiating a glucagon-like peptide-1 receptor agonist: a retrospective analysis of the Diabetes Prospective Follow-Up Registry. Diabetes Obes Metab.

[20] Palmer SC, Tendal B, Mustafa RA, Vandvik PO, Li S, Hao Q, Tunnicliffe D, Ruospo M, Natale P, Saglimbene V, Nicolucci A, Johnson DW, Tonelli M, Rossi MC, Badve SV, Cho Y, Nadeau-Fredette AC, Burke M, Faruque LI, Lloyd A, Ahmad N, Liu Y, Tiv S, Millard T, Gagliardi L, Kolanu N, Barmanray RD, McMorrow R, Raygoza Cortez AK, White H, Chen X, Zhou X, Liu J, Rodriguez AF, Gonzalez-Colmenero AD, Wang Y, Li L, Sutanto S, Solis RC, Diaz Gonzalez-Colmenero F, Rodriguez-Gutierrez R, Walsh M, Guyatt G, Strippoli GFM (2021). Sodium-glucose cotransporter protein-2 (SGLT-2) inhibitors and glucagon-like peptide-1 (GLP-1) receptor agonists for type 2 diabetes: systematic review and network meta-analysis of randomised controlled trials. BMJ.

[21] Overgaard RV, Hertz CL, Ingwersen SH, Navarria A, Drucker DJ (2021). Levels of circulating semaglutide determine reductions in HbA1c and body weight in people with type 2 diabetes. Cell Rep Med.

[22] Russo G, Di Bartolo P, Candido R, Lucisano G, Manicardi V, Giandalia A, Nicolucci A, Rocca A, Rossi MC, Di Cianni G (2023). The AMD ANNALS: a continuous initiative for the improvement of type 2 diabetes care. Diabetes Res Clin Pract.

[23] Vitale C, Rosano GM, Prasad K (2016). Need for streamlined use of DPP-4 inhibitors in the treatment of type 2 diabetes. Cardiovasc Diabetol.

